# Androgenesis of Red Cabbage in Isolated Microspore Culture In Vitro

**DOI:** 10.3390/plants10091950

**Published:** 2021-09-18

**Authors:** Anna Mineykina, Ludmila Bondareva, Alexey Soldatenko, Elena Domblides

**Affiliations:** Federal State Budgetary Scientific Institution Federal Scientific Vegetable Center (FSBSI FSVC), VNIISSOK, 143072 Moscow Region, Russia; lyuda_bondareva@mail.ru (L.B.); alex-soldat@mail.ru (A.S.)

**Keywords:** red cabbage, microspore culture, abnormal misshapen embryos, DH plants, homozygous lines, induction of embryogenesis, regeneration, ploidy level

## Abstract

Red cabbage belongs to the economically important group of vegetable crops of the Brassicaceae family. A unique feature of this vegetable crop that distinguishes it from other members of the family is its unique biochemical composition characterized by high anthocyanin content, which gives it antioxidant properties. The production mainly uses F1 hybrids, which require constant parental lines, requiring 6–7 generations of inbreeding. Culture of isolated microspores in vitro is currently one of the promising methods for the accelerated production of pure lines with 100% homozygosity. The aim of this study is to investigate the factors and select optimal parameters for successful induction of red cabbage embryogenesis in isolated microspore culture in vitro and subsequent regeneration of DH plants. As a result of research, for the first time, it was possible to carry out the full cycle of obtaining DH plants of red cabbage from the induction of embryogenesis to their inclusion in the breeding process. The size of buds containing predominantly microspores at the late vacuolated stage and pollen at the early bi-cellular stage has to be selected individually for each genotype, because the embryoid yield will be determined by the interaction of these two factors. In the six samples studied, the maximum embryoid yield was obtained from buds 4.1–4.4 mm and 4.5–5.0 mm long, depending on the genotype. Cultivation of microspores was carried out on liquid NLN culture medium with 13% sucrose. The maximum number of embryoids (173.5 ± 7.5 pcs./Petri dish) was obtained on culture medium with pH 5.8 and heat shock at 32 °C for 48 h. Successful embryoid development and plant regeneration by direct germination from shoot apical meristem were achieved on MS culture medium with 2% sucrose and 0.7% agar, supplemented with 6-benzylaminopurine at a concentration of 1 mg/L. Analysis of the obtained regenerated plants, which successfully passed the stage of adaptation to ex vitro conditions by flow cytometry, showed that most of them were doubled haploids (up to 90.9%). A low number of seeds produced by self-fertilization in DH plants was observed.

## 1. Introduction

Red cabbage (*Brassica oleraceae* L. convar. *capitata* (L.) Alef. var. *rubra* (L.) Thell.) is one of the valuable crops that belong to the same botanical species as white cabbage, sharing the same origin. The value of this crop is its balanced biochemical composition, which contains potassium, vitamins B and PP, ascorbic acid, carotene, and organic acids [1]. Its uniqueness and difference from other types of cabbage crops, according to Borisov et al. [2], is the high content of essential amino acids, especially arginine, histidine, methionine, phenylalanine, and tryptophan. The purple coloring of the leaves of this cabbage is due to the increased content of the coloring pigment, anthocyanin, which can reach 204–500 mg/% [2]. Due to the anthocyanin content, red cabbage has strong antioxidant and anti-inflammatory effects and removes heavy metal salts and toxins [3,4]. Grape and red cabbage are leaders among fruits and vegetables in terms of anthocyanin content [5]. Currently, there is an increased interest in good nutrition and in the inclusion of foods rich in biologically active substances in the human diet, including red cabbage.

This crop is characterized by high yields, transportability and high storage quality, and the availability of varieties with different maturity dates makes it possible to have fresh produce throughout the year. F1 hybrids are now widespread, with high yields, easy maturity, uniformity of plants, resistance to major diseases, and suitability for mechanized harvesting. The expansion of the assortment of red cabbage by creating high-yielding and leveled F1 hybrids (with high marketable quality, increased content of biologically active substances, with different maturity dates, resistance to diseases and pests) in the shortest possible time is very relevant.

Since red cabbage is a cross-pollinated plant, constant parental lines are required to produce hybrids. In order to create inbred lines using traditional methods, it is necessary to carry out their forced self-pollination for 6–7 generations, which significantly increases the duration of the breeding process and the cost of obtaining F1 hybrids.

Currently, biotechnological methods are widely used in cabbage crop breeding. Among them, the most promising is the method of isolated microspore culture in vitro to create doubled haploid lines (DH-lines) [6]. This method makes it possible to create homozygous lines within one generation and promotes the expansion of genetic recombinant forms, including those with recessive traits. The basic technology protocol for producing doubled haploids using isolated microspore culture in vitro, developed in the early 1980s [7] for rapeseed, was subsequently adapted by scientists for different members of the genus *Brassica* L. [8,9,10]. In the Russian Federation, this technique has been successfully applied to members of the Brassicaceae family, and F1 hybrids based on doubled haploid cabbage lines of white cabbage, Chinese cabbage, broccoli, and kohlrabi were obtained [11,12,13,14,15,16,17,18,19,20]. As early studies show, the protocol is not standard and needs to be adapted to the specific species and genotype. There is no information in the literature on the successful use of isolated microspore culture to produce doubled haploids of red cabbage; therefore, optimization of the protocol and obtaining DH plants is relevant and was the aim of this study.

## 2. Results

### 2.1. Development of Red Cabbage Embryoids from Microspores in In Vitro Culture

**Figure 1:**
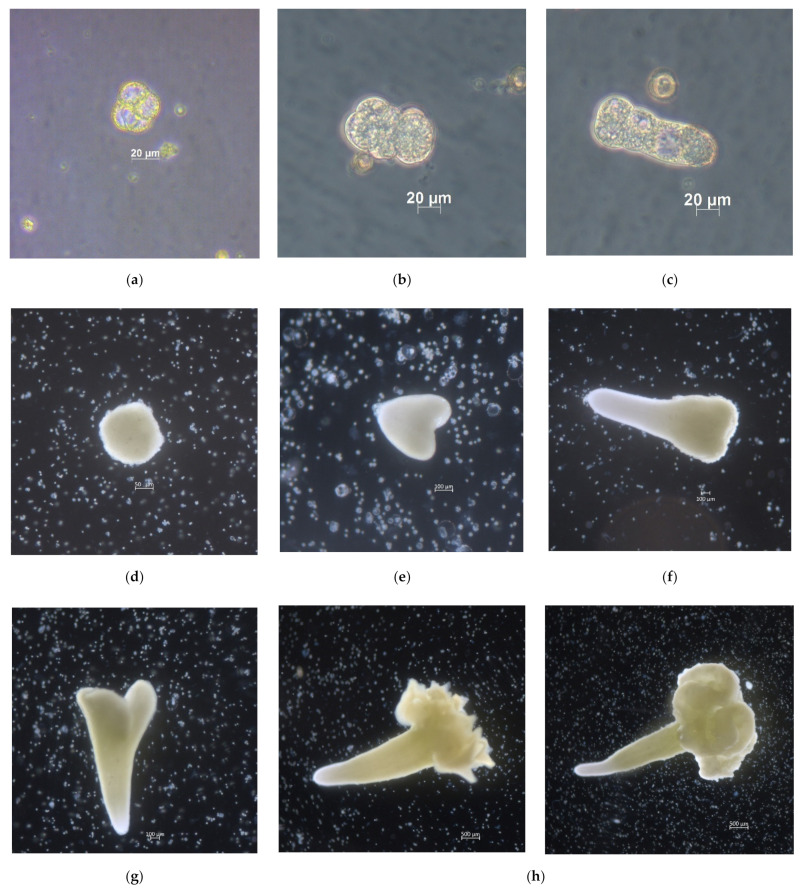
Microspore division of red cabbage and embryoid development stages: (**a**) first divisions in microspore culture (3 days); (**b**) microspore divisions on day 10 of cultivation; (**c**) suspensor-like structure; (**d**) embryoid at the globular stage of development; (**e**) embryoid at the heart stage of development; (**f**) embryoid at the torpedo stage; (**g**) early cotyledon stage; (**h**) embryoids with overgrown cotyledons of various morphologies.

The first divisions in the red cabbage microspore culture were observed after three days of cultivation (Figure 1a). Further division of microspores proceeded in two directions, either by direct development of embryoids (Figure 1b) or by the formation of suspensor-like structures (Figure 1c). After 14 days, most embryoids had reached the globular stage of development and were visible to the naked eye (Figure 1d). By 30 days of cultivation, the embryoids had reached the cotyledon stage (Figure 1g), ready to be transferred to solid culture medium for plant regeneration. In some embryoids, cotyledon outgrowth of different morphologies was observed (Figure 1h).

### 2.2. Determination of the Dependence of the Yield of Embryoids on the Size of the Buds

It is known from the literature that *Brassica* plants are capable of embryogenesis of late vacuolated stage microspores and early two-celled pollen grains. The optimal bud size corresponding to these stages varies greatly in cabbage crops [21,22].

In the experiment to determine the optimum size of the red cabbage buds, the protocol parameters developed earlier for cabbage crops were used [18,23].

As a result, a wide variation in the optimum size of red cabbage buds containing responsive microspores for embryogenesis induction was confirmed, and an intraspecific genotypic relationship was shown (Table 1).

**Figure 2:**
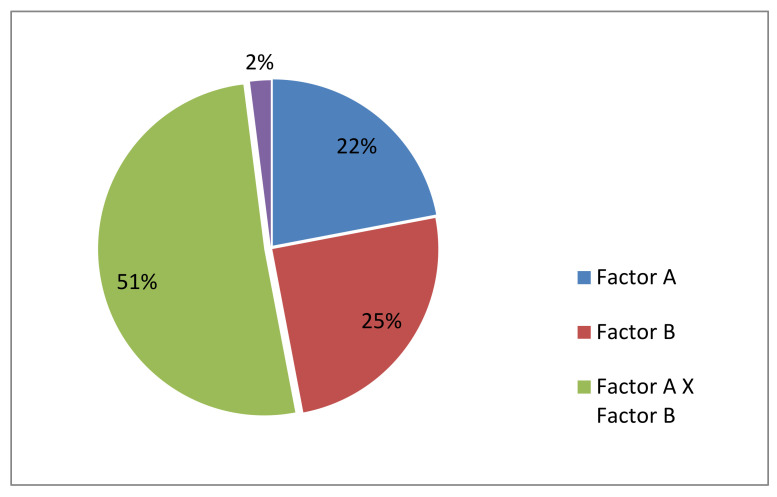
Contribution of the factors «bud size» (**A**) and «genotype» (**B**) and the interaction of respective factors on in vitro embryogenesis induction of red cabbage in microspore culture in vitro. Note: ANOVA and Fisher test were used. Significant difference: Factor A: F_observed_99.01 > F_theor._3.10; Factor B: F_observed_84.02 > F_theor._2.87; A × B: F_observed_55.79 > F_theor._2.28.

Factor analysis found a significant effect of bud size and genotype on embryoid yield, with an influence fraction of 22% and 25%, respectively. Since embryoid yield is mainly determined by the interaction of these factors (51%), the bud size ranges most responsive to embryogenesis induction were taken individually for each genotype for a further series of experiments (Figure 2).

### 2.3. Influence of Acidity of the Culture Medium on Embryoid Yield

The use of culture media with different levels of acidity has shown that the pH value of the culture medium has a significant effect on the yield of red cabbage embryoids.

**Figure 3:**
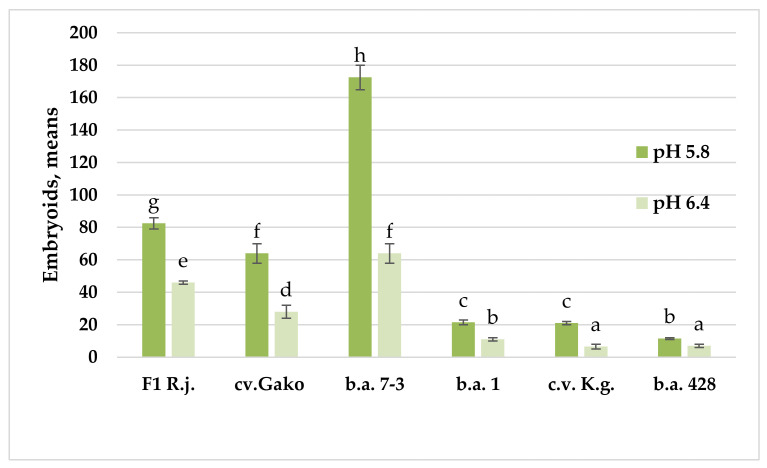
Influence of acidity of the culture medium on the induction of embryogenesis in red cabbage. Note: Values presented are means of three independent experiments with five replicates in each ± SE (standard error). One-way analysis of variance (ANOVA) was used, and means were compared using a Student’s *t* test with a probability of 95%. Values marked with a similar letter had no significant differences at *p* ≤ 0.05. Embryos visible to the naked eye were counted in a Petri dish (6 cm in diameter) at 30 days of cultivation.

The most optimal culture medium for all genotypes was pH 5.8. Reducing the acidity of the culture medium to pH 6.4 almost halved the embryoid yield. No genotypic differences were found in the red cabbage samples studied. The trend toward a reduction in embryoid yield with decreasing acidity can be seen in all samples, irrespective of the degree of genotype responsiveness (Figure 3).

### 2.4. Heat Shock Treatments

Heat shock treatment for 24–48 h at 32 °C was most effective for the induction of embryogenesis in responsive red cabbage genotypes: cv. Gako, b.a. 7-3, and F_1_ Red Jewel. At the same time, the number of formed embryoids increased significantly with the duration of heat treatment of microspores up to 48 h. The maximum number of embryoids was obtained in genotype cv. Gako and amounted to 131.5 ± 16.5 per Petri dish, which was significantly higher than the number of embryoids formed in heat shock treated for 24 h.

**Figure 4:**
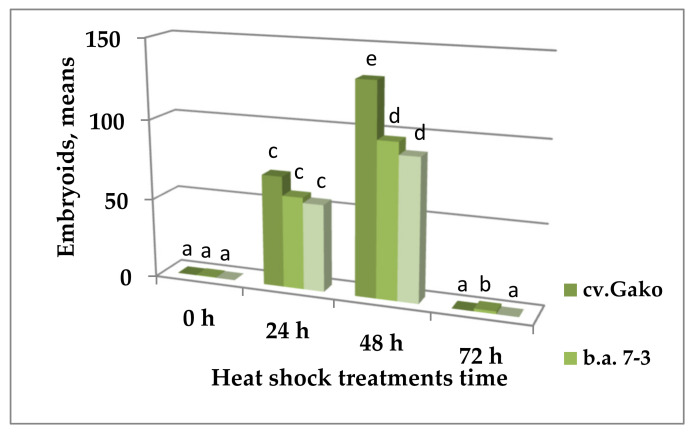
Influence of the duration of heat shock treatment of the in vitro culture microspores at 32°C on embryoid yield in responsive genotypes of red cabbage. Note: Values presented are means of three independent experiments with five replicates in each ± SE (standard error). One-way analysis of variance (ANOVA) was used, and means were compared using a Student’s *t* test with a probability of 95%. Values marked with a similar letter had no significant differences at *p* ≤ 0.01. Embryoids visible to the naked eye were counted in a Petri dish (6 cm in diameter) at 30 days of cultivation.

**Figure 5:**
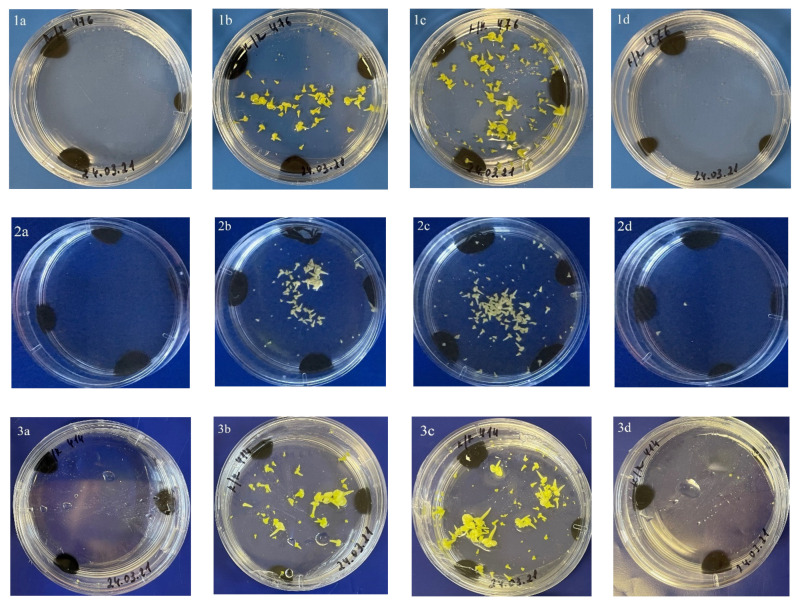
Embryoid yield as a function of the duration of heat shock treatment at 32 °C in the genotypes of red cabbage: 1—cv. Gako (476), 2—b.a. 7-3, 3—F_1_ Red Jewel (414); a—control (without temperature shock), b—24 h, c—48 h, d—72 h.

Heat shock duration for 72 h inhibited embryoid development. In the variants where microspores were not heat shock treated at 32 °C but incubated immediately at 25 °C, no embryoids were formed (Figure 4 and Figure 5).

### 2.5. Plant Regeneration

**Figure 6:**
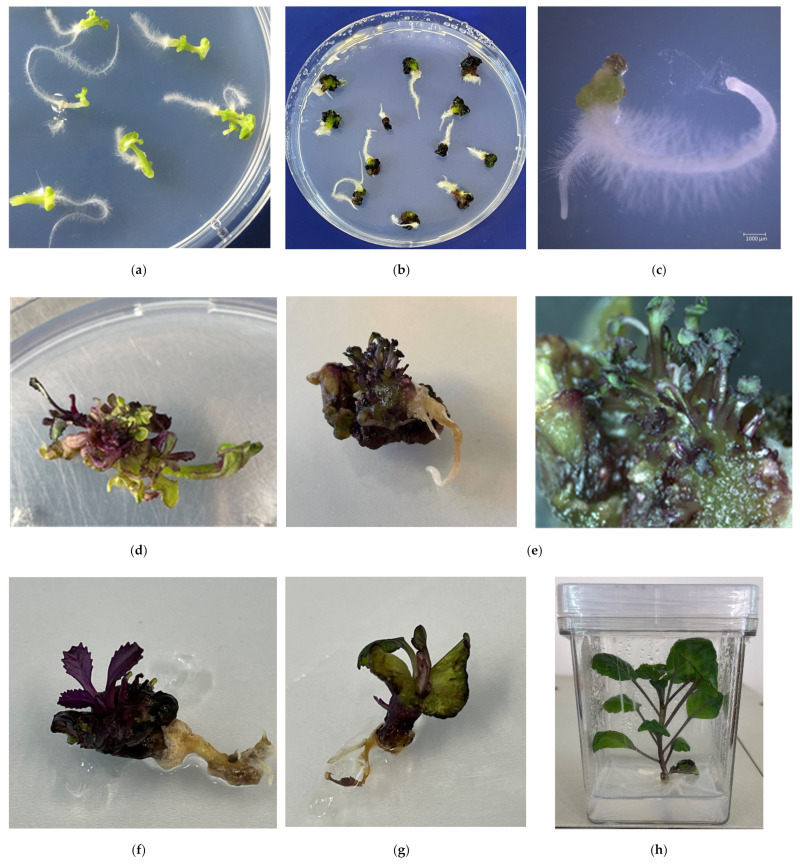
Features of embryoid of red cabbage development on solid MS culture medium: (**a**,**b**) Embryoids on solid MS culture medium; (**c**) Embryoid dedifferentiated into callus; (**d**) The formation of secondary shoots from callus; (**e**) The formation of multiple secondary shoots from overgrown hypocotyl tissue; (**f**,**g**) Direct germination from the shoot apical meristem; (**h**) Rooting shoots on solid MS culture medium.

When the cotyledon stage of development was reached, normally developed embryoids were transferred to solid MS culture medium without growth regulators. The embryoids greatly increased in size, turned green and formed a root (Figure 6a) within a week. As they grew, the embryoids accumulated anthocyanins of varying intensity (Figure 6b). Most of the shoots were derived from tissues of the expanded hypocotyl or cotyledons. Some embryoids were dedifferentiated into a callus during regeneration, on which multiple secondary shoots were subsequently formed (Figure 6c–e). In case of poor embryoid development and lack of shoot formation, they were transferred to solid MS culture medium supplemented with BAP (1 mg/L) or BAP (1 mg/L) and GA (0.1 mg/L). The use of culture medium supplemented with BAP at the concentration of 1 mg/L promoted the development of 12–25% of embryoids by direct germination from the shoot apical meristem (Figure 6f,g). Addition of gibberellic acid to the culture medium had no significant effect on direct shoot germination, but like BAP, promoted the formation of growth points and shoots in poorly developing embryoids.

**Figure 7:**
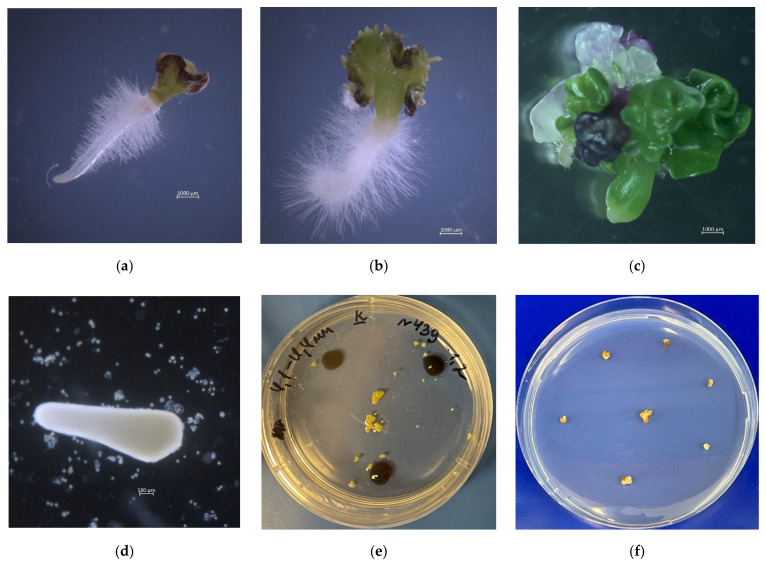
Peculiarities of abnormal development of red cabbage embryoids: (**a**) normally developed embryoid on solid MS culture medium; (**b**) formation of secondary shoots on overgrown cotyledons; (**c**) distorted leaves; (**d**) lack of apical structures in developing embryoid; (**e**) morphogenetic development disorder of embryoid of cv. Kamennaya golovka on NLN culture medium; (**f**) developed globular structures of cv. Kamennaya golovka on solid MS culture medium.

Among the red cabbage accessions, genotype cv. Kamennaya golovka was observed to produce abnormal misshapen embryos, in which the genetic programs of axial frame formation and its subsequent differentiation into functional organs (cotyledon, hypocotyl, root) were disrupted (Figure 7c,d). Embryoid development was suppressed, and globular structures were formed (Figure 7e), or the embryoids consisted of a shortened hypocotyl without any apical structures (Figure 7d). On solid culture medium, such embryoids failed to regenerate plantlets, underwent necrosis, and subsequently died (Figure 7f). The use of cultural media with BAP and GA for such abnormal embryoids was also unsuccessful, and no plants were obtained.

As well-developed shoots formed, they were transplanted to fresh nutrient medium for rooting. A 100% rooting rate on MS culture medium without growth regulators with 2% sucrose was observed for the developed red cabbage plantlets (Figure 6h).

Red cabbage is a biennial crop and requires a vernalization stage (at 6° C for 70 days) to produce flowering plants. Since it is not always possible to plant regenerated plants in the field in the current season (for countries with negative winter temperatures), as they may not reach the necessary phase (15–20 leaves) for successful vernalization, it is quite often necessary to keep the plants in vitro until the next season. In our experiment, we also encountered this problem. All regenerated plants that had not formed a well-developed root system in in vitro culture by 15 July were left until the next season and every 4 weeks were transferred to solid MS culture medium without growth regulators with 2% sucrose.

In all, 95 plants derived from five different genotypes (from cv. Kamennaya golovka plantlets were not received) that had formed a well-developed root system by mid-July were planted in the soil to adapt to in vivo conditions. Of these, 88 plants were successfully acclimatized and evaluated for ploidy levels before planting in the field (Table 2).

### 2.6. Evaluation of the Regenerated Plants of Red Cabbage

**Figure 8:**
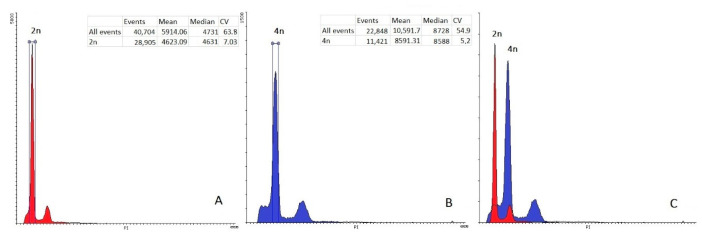
Flow cytometry histograms of red cabbage plants obtained from isolated microspore culture in vitro. External control samples were analyzed separately, without changing the cytometer settings. (**A**) The diploid (2n = 2x = 18) plant of red cabbage as a control; (**B**) tetraploid regenerated plant; (**C**) histogram of both the diploid control and tetraploid sample.

In total, the ploidy of 88 red cabbage plants, obtained through isolated microspore culture and successfully passed the stages of adaptation to in vivo conditions, was analyzed for ploidy level using flow cytometry. Most of them, i.e., 80 (90.9%) were diploids, 5 (5.7%) were tetraploids, and 3 (3.4%) were aneuploids (Table 2, Figure 8). DH and tetraploid plants could be included in the breeding process without the additional step of chromosome doubling by colchicinization.

To determine whether DH plants could be included in further breeding programs, they were subjected to self-pollination to produce seed progeny and determine the degree of self-incompatibility. As a result, a low degree of seed setting by geitonogamous pollination in buds was observed in self-incompatible plants. On average, this index ranged from 1 to 5 seeds/stem. Consequently, when breeding DH plants, the fact of low seed setting in pods should be taken into account, and more pods should be pollinated in order to maintain and preserve seed progeny.

**Figure 9:**
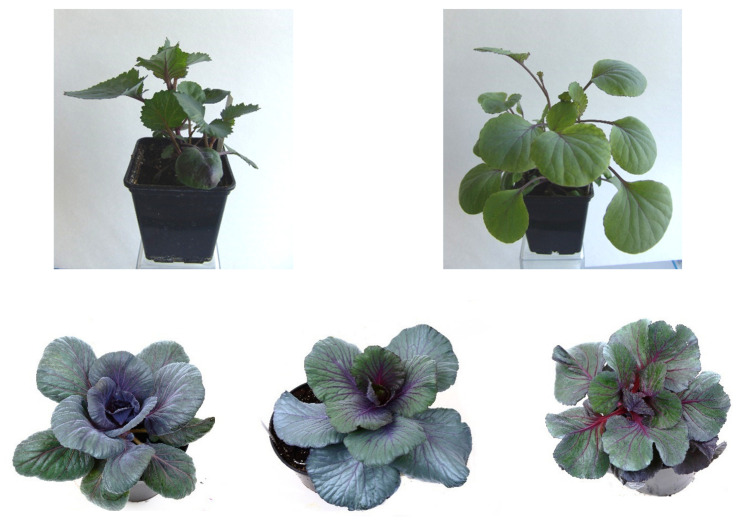
Genotypic diversity of the obtained regenerated plants of red cabbage under in vivo conditions b.a. 7-3.

Visual assessment of doubled haploid plants by morphological traits within each variety revealed a wide range of morphogenetic diversity in the resulting lines (Figure 9).

## 3. Discussion

Work on the morphogenetic potential for cabbage regeneration was initiated in 1975 by Bajaj and Nietsch [24]. In their experiments with in vitro tissue culture method, they showed the influence of genotype and culture medium components on callus formation, morphogenesis, and plant regeneration. A successful protocol for rapid and efficient plant regeneration from protoplasts was developed for red cabbage [25]. Pavlovic et al. [26] and Al-Hardan et al. [27] studied somatic embryogenesis of cabbage crops, including red cabbage, from different types of explants. Cilingir et al. [28] for the first time studied androgenesis of red cabbage in anther culture. However, in these studies, the yield of the resulting plants was negligible. As reported in the study of Vyvadilova et al. [29], in the study of embryogenesis in microspore culture in commercial varieties of *Brassica oleraceae*, the authors managed to induce microspore division in all tested species. However, the authors do not report the successful development of embryoids and obtaining DH plants of red cabbage.

As seen in early studies, the success of biotechnological methods requires an efficient and reliable protocol. Since many authors note high genotypic specificity, such protocols need to be adapted to a particular species, variety, or genotype.

To the best of our knowledge, there are no reports of embryo-derived, subsequent regeneration of cabbage plants in isolated microspore culture in vitro.

Based on our experience with isolated microspore culture in vitro with Brassicaceae plants, we conducted a comprehensive experiment to optimize the methodology for successful induction of cabbage embryogenesis in isolated microspore culture in vitro with subsequent regeneration into plants.

Numerous factors are known to influence the process of embryogenesis in cabbage crops, the optimum values of which are decisive for the efficiency of the isolated microspore culture protocol in vitro. Factors such as cultivation conditions of donor plants, genotype, stage of development of microspores [7,21,22,30], type of pretreatment of buds and microspores [21,31], culture media composition, and cultivation conditions are subjects of ongoing research and modifications [6,8,32]. A detailed comparative analysis of most factors influencing androgenesis in *Brassica* L. species in microspore culture in vitro is presented in the review article by Shmykova et al. [33].

Among the many potential factors for optimization, several key ones were selected, including bud size, culture medium acidity level, and heat shock. This group of factors is stressful for switching microspores from gametophytic to sporophytic development. At the same time, for different representatives of the genus *Brassica*, the obtained indicators of these factors in the literature are contradictory. This is a prerequisite for their optimization, including red cabbage.

As noted in early studies on the efficiency of androgenesis in cabbage crops, the cultivation success is largely determined by the stage of development of microspores of donor plants capable of division and further embryogenesis [21,34]. For the genus *Brassica*, a close correlation between the stage of microspore development and bud size has been shown, and optimal sizes of responsive buds for embryogenesis have been determined, depending on the species [7,22,30]. Our study for red cabbage showed wide differences in the number of embryoids formed depending on bud length in different genotypes. Potentially embryogenic microspores in red cabbage, depending on the genotype, were shown to be within a wide range of bud lengths from 3.7 to 5.7 mm. In early studies, it was observed that the size range of buds capable of embryogenesis even on different inflorescences of the same plant varies [21]. It is also known that cabbage buds of the same size simultaneously contain microspores at different developmental stages, but the majority of microspores are distributed at two nearest stages of development. More suitable for androgenesis are buds in which most of the microspores are at the late vacuolized stage, and a small number of early two-celled pollen may be present. In our experiment, the results of earlier studies were confirmed. Dividing buds into narrower length ranges allowed us to more clearly identify potentially embryogenic microspores in individual genotypes, as well as increase the embryoid yield and speed up the work considerably.

The optimum pH value of the culture medium for in vitro cultivation of cabbage plants was found in early studies to be within 5.0–5.8 [35,36]. More recent studies have shown the effectiveness of cabbage embryogenesis induction when pH values are increased to 6.2–6.4 [37,38]. However, if the pH in the culture medium was lower or higher than 5.5–5.8, it reduced the embryoid yield in broccoli [35]. Yuan et al. [38] showed that in non-responsive genotypes, no embryoids were formed on culture media with a pH of 5.8. Increasing the pH to 6.2–6.4 in the study was found to be crucial to the successful induction of microspores.

For red cabbage, the pH range of the culture medium chosen for the experiment was not the decisive factor in the initiation of embryogenesis. At both pH values of 5.8 and 6.4, embryoids were formed in all genotypes. However, increasing the pH of the culture medium significantly reduced the number of formed embryoids in all genotypes by almost twofold. Barinova et al. [39], when investigating the mechanism of action of high pH on the cultivation of tobacco and snapdragon microspores, came to the conclusion that high pH value disturbs the sucrose breakdown and decreases its uptake by microspores, leading to their death. The authors showed that the maximum invertase activity in microspores was at pH 5.0.

Heat shock is an effective stressor for switching from gametophytic to sporophytic development of microspores, especially for *Brassica* cultures [30,31,40]. The effectiveness of temperature treatment of microspores during cultivation depends not only on the temperature but also on the timing of exposure. Heat regimes of 32 to 40 °C with varying time exposures (1 to 10 days) are most commonly used for this purpose.

Su et al. [41] studied the mechanism of short-term heat shock treatment in *Brassica oleraceae* at the molecular level at 32 °C and as a control at 25 °C for 24 h. It was shown that no embryoids were formed without heat shock. The comparative proteomic analysis performed by the authors suggested that the induction of cabbage embryogenesis by high temperature might be related to carbohydrate metabolism. Upregulated proteins activated during heat shock provide the necessary energy for microspores during early embryogenesis.

For red cabbage, as well as for some *Brassica* species, a heat shock treatment at 32 °C for 24 h was found to be effective for the successful induction of embryogenesis. The data are comparable to earlier studies showing the effectiveness of embryogenesis induction in cabbage after an appropriate heat shock [38,42]. However, our results showed that increasing the time of exposure to elevated temperatures to 48 h promoted greater embryoid formation in red cabbage. However, a prolonged heat shock for 72 h was critical for microspore division and completely inhibited microspore development. Similar results were observed by Pang et al. [43] in the cultivation of cauliflower anthers.

In early studies on the regeneration of *Brassica* and other plant species, it was shown that the ability to regenerate is genetically controlled. Different genotypes require different cultivation conditions [44,45]. Pavlovic et al. [26] observed a high regeneration rate of somatic cabbage embryoid shoots from hypocotyl explants on MS culture medium containing different concentrations of hormones. In a more recent study, Al-Hardan et al. [27] also studied the effect of culture medium and hormone concentration on direct somatic embryogenesis of cabbage using different types of explants (cotyledon, hypocotyl, and leaf). The result was developed plants from cotyledon explants with a 45% conversion rate on B5 culture medium with a lower hormone concentration.

Regeneration of red cabbage embryoids into plants occurred in several ways: dedifferentiation into callus, formation of secondary shoots from hypocotyl or cotyledon epidermis, and direct germination from the shoot apical meristem. These pathways of embryoid development have been studied previously and are characteristic of cabbage crops [46,47,48]. It should be noted that direct shoot germination is the most desirable and effective pathway for the rapid development of regenerated plants. Obtaining plants through callus and secondary shoots is labor intensive and requires several stages of subculturing on fresh culture medium [47].

In the present study, the effects of gibberellic acid and BAP on direct germination from the apical meristem were studied. In tissue culture, gibberellic acid promotes shoot stem elongation. However, for red cabbage, its effective effect on direct germination was not observed. On the contrary, the use of culture medium supplemented with BAP promoted 12–25% of embryoids to form shoots by direct germination from the apical meristem. In addition, genotypic features of embryoid formation and development were observed. Some red cabbage genotypes formed abnormal embryoids with deformed and enlarged cotyledons. Such embryoids failed to develop, underwent necrosis, and died. Hadfi et al. [49] studied auxin-mediated processes in the developing embryoid of *Brassica juncea*. The authors showed that morphogenetic changes in early globular embryoids are associated with impaired auxin transport, which leads to changes in the polarity of the apical–basal axis. This genetic feature of embryoid development is of interest and requires a separate study.

Determining the ploidy of the regenerated plants is an indispensable element in the technology of doubled haploids because the resulting regenerated plants have different ploidy levels [50,51]. The occurrence of spontaneously doubled regenerated plants is characteristic and has been studied in the genus *Brassica* [52,53]. The results of ploidy determination by flow cytometry showed that most of the red cabbage plants adapted to in vivo conditions were doubled haploids (up to 90.9%). Lack of haploids among the plants analyzed is probably due to their low viability and their elimination at the stage of adaptation. The high frequency of chromosome doubling in DH plants avoids the colchicine stage.

In earlier studies on the incorporation of broccoli DH plants into the breeding process, the authors observed a low seed set in pods under heterogamous pollination. In a study by Farnham [54], the average number of seeds varied from 2.4 to 5.8 pcs/stem for annual inbreeding, depending on the genotype, and from 1.8 to 3.5 pcs/stem for DH lines. According to Zablotskaya et al. [55], the average number of seeds at geitonogamous pollination in all DH broccoli lines was 4.91 pcs/stem. All this serves as a prerequisite for further study of this index for the suitability of the obtained lines of doubled haploids in breeding work. Red cabbage also showed a low degree of seed setting in the pods at self-pollination.

## 4. Materials and Methods

### 4.1. Plant Material and Plant Growing Conditions

Six accessions of cabbage from the cabbage breeding and seed production laboratory of the of the Federal State Budgetary Scientific Institution Federal Scientific Vegetable Center (FSBSI FSVC) were used for the evaluation for embryogenesis responsiveness.

Donor plants were grown in an environmental chamber at 16 h day/8 h night, 19 °C, and 9000 lux illumination. Buds were collected from plants of each variety at the same phase of plant development (initial stage of flowering).

### 4.2. Influence of Size of Buds on Embryoids Yield

The differential staining technique [56] was used to determine the stage of development of microspores, and an Axio Imager A2 with an AxioCam MRc5 camera (Carl Zeiss Microscopy GmbH, Jena, Germany) was used for imaging. Only buds containing microspores at the late vacuolated microspore stage and pollen at the early bi-cellular stage were used. Because potentially embryogenic microspores in red cabbage covered a wide range depending on bud length, buds of each genotype were divided into groups for the experiment: 3.7–4.0 mm; 4.1–4.4 mm; 4.5–5.0 mm; 5.1–5.7 mm. Factorial ANOVA and Fisher test were used to determine the effect of genotype and bud size on embryo yield.

### 4.3. Microspore Culture

The technique of cultivation of Brassicaceae family microspores developed earlier in the laboratory of biotechnology (FSBSI—Federal Scientific Vegetable Center, Moscow, Russia) was used [23,57,58].

Buds were separated from young inflorescences, placed in Petri dishes with filter paper dampened with distilled water, and pre-cold treated at 6 °C for 1 day. The buds were sterilized in 96% ethanol for 30 s and in 5% aqueous sodium hypochlorite solution for 15 min with the addition of Tween 20 (1 drop per 100 mL of solution). The buds were then washed three times in distilled sterile water for 10 min. All liquid media were sterilized using a bottle-top vacuum polyether sulfone (PES) filter system with a pore diameter of 0.22 μm (Corning, NY, USA). Grade II sucrose was used to prepare NLN-13 [7] washing culture medium and Grade I sucrose (Sigma, St. Louis, MO, USA) was used for NLN-13 cultivation culture medium. The sterile buds were crushed in NLN-13 washing culture medium (30 buds per 6 mL of medium). The resulting microspore suspension was filtered through a 40 µm pore diameter nylon filter into a 15 mL Falcon sterile test tube. The suspension was then centrifuged for 5 min at 130 *g* at 4 °C. The supernatant was gently removed, and NLN-13 washing culture medium was added again. This step was repeated twice, and then liquid NLN-13 culture medium (pH 5.8 to 6.4 depending on the experiment) was added to the washed microspores. Microspore suspension density was determined using a Goryaev counting chamber (MiniMed, Bryansk, Russia). For incubation, a microspore suspension of density 2 × 10^4^ mL^−1^ was used. Briefly, 5 mL of microspore suspension was poured into sterile Petri dishes, 60 mm in diameter (Biomedical Ltd., Moscow, Russia). A 1% suspension of activated carbon in 0.5% agarose was preliminarily added to the Petri dishes. Petri dishes with microspore suspension were then sealed and placed in the incubator Binder BF 260 BINDER GmbH (Tuttlingen, Germany) with different temperatures (25 and 32 °C) for heat shock treatments continuously for 24, 48, and 72 h. Cultivation was carried out in the dark using a PSU-10i shaker platform (Biosan) at a rate of 40 shakes/minute. Microspore culture was monitored every 3–7 days using a Primo Vert inverted microscope (Carl Zeiss Microscopy GmbH, Jena, Germany) and a Stemi 508 stereomicroscope. Embryogenesis was photodocumented using an Axiocam 305 color camera (Carl Zeiss Microscopy GmbH, Jena, Germany).

### 4.4. Influence of Acidity of the Culture Medium on Embryoid Yield

Microspores were cultured in NLN-13 medium supplemented with 13% (*w/v*) sucrose at pH 5.8 or 6.4. Three replicates were used for each experimental variant. The experimental data were processed using the standard Microsoft Excel 2010 application software package. Statistical analysis was performed using one-way analysis of variance (ANOVA), and means were compared using a Student’s *t* test with a probability of 95%. The statistical analyses were carried out using Statistica 8.0 (Statsoft, www.statsoft.com accessed on 5 July 2021).

### 4.5. Influence of the Duration of Heat Shock Treatment on Embryoid Yield

Highly responsive genotypes according to previous studies cv. Gako, b.a. 7-3, and F1 Red Jewel. Microspores were cultured in NLN-13 medium supplemented with 13% (*w/v*) sucrose at pH 5.8 at 32 °C temperature shock for 24, 48, and 72 h. As a control, microspores were cultured immediately at 25 °C. Three replicates were used for each experimental variant. The experimental data were processed using the standard Microsoft Excel 2010 application software package. Statistical analysis was performed using one-way analysis of variance (ANOVA), and means were compared using a Student’s *t* test with a probability of 95%. The statistical analyses were carried out using Statistica 8.0 (Statsoft, www.statsoft.com accessed on 5 July 2021).

### 4.6. Plant Regeneration

As soon as embryoids reached the cotyledon stage of development, they were transferred to solid MS culture medium [59] with 2% sucrose (Grade II grade) and 0.7% agar (Sigma), pH 5.8. If embryoids were poorly developed, they were placed on solid MS culture medium supplemented with BAP (1 mg/L) or BAP (1 mg/L) and GA (0.1 mg/L) for 10–14 days. Growing shoots were dissected and placed on solid MS culture medium without growth regulators with 2% sucrose for rooting. In the case of long-term preservation in in vitro culture, the microshoots were transplanted to fresh nutrient medium every 4 weeks. Cultivation was performed on the shelves under mixed light of two types of fluorescent lamps: OSRAM Fluora L36W/77 (mainly blue and red spectrum) and Philips 36W/54-765 (mainly white spectrum), at a total illumination of 3000 lux at 16 h day/8 h night and 24 ± 2 °C.

### 4.7. Adaptation of Regenerated Plants to In Vivo Conditions

The rooted shoots with 5–6 leaves were planted in culture vessels (1 liter) with a mixture of peat–perlite (7:3) and covered with plastic cups with top perforations for 10 days to allow the stomata to adapt to lower humidity.

The pots were placed in a growth chamber at 21 °C, a 16 h day/8 h night photoperiod, and 8000 lux illumination (Plantastar HPS lamp, 600 W, Osram). Plants were watered as needed and fertilized once a week.

### 4.8. Ploidy Level Determination

Relative DNA content was determined by flow cytometry techniques using propidium iodide and a Partec CyFlow PA flow cytometer (Partec, GmbH, Münster, Germany) with a 532 nm laser source. Approximately 20 mg of young leaves was cut with a razor blade in 1 mL of lysis buffer (0.2 M Tris, 4 mM MgCl_2_, 50 µg/mL RNA-ase, 0.5% (v/v) TRITON X-100, 0.5% (*v/w*) polyvinylpyrrolidone K15, and 50 µg/mL propidium iodide, pH 7.5) [60]. Samples were filtered through a 50 μm nylon filter. The diploid (2n = 2x = 18) donor plants of red cabbage cv. Gako were used as external standards to determine the ploidy.

### 4.9. Producing Seed Offspring of DH Plants

DH plants were grown in the field until they reached the stage of “stalking” (15–20 true leaves). When the plants reached the desired stage, they were subjected to vernalization at 6 °C for 70 days. After vernalization, plants were transplanted into the field to produce flowering stems. At the flowering stage, plants were subjected to geitonogamous pollination of buds and flowers to determine the degree of self-incompatibility. It was determined by seed setting in pods during flower self-pollination: high-setting 0–1 pcs/stem, medium-average 2–5 pcs/stem, low or no-more than 5 pcs/stem [61].

## 5. Conclusions

In this study, for the first time, a complete cycle of DH production of cabbage plants in isolated microspore culture in vitro was carried out. As a result, key culture inducing factors were studied and optimal parameters for increasing embryoid yield and plant regeneration.

## Figures and Tables

**Table 1:** Embryoid yield* in different accessions of red cabbage as a function of bud size.

Genotype	Bud Length, mm			
	3.7–4.0	4.1–4.4	4.5–5.0	5.1–5.7
cv. Kamennaya golovka	1.0 ± 1.5 ^a^	23.0 ± 4.0 ^d^	4.5 ± 3.5 ^ab^	0 ^a^
b.a. 428	8.5 ± 1.5 ^b^	14.0 ± 2.0 ^c^	0 ^a^	0 ^a^
b.a. 1	0 ^a^	11.5 ± 0.5 ^c^	21.5 ± 1.5 ^d^	0 ^a^
cv. Gako	0 ^a^	64.0 ± 6.0 ^e^	131.5 ± 16.5 ^h^	0 ^a^
F_1_ Red Jewel	0 ^a^	82.5 ± 3.5 ^f^	4.5 ± 0.5 ^b^	0 ^a^
b.a. 7-3	0 ^a^	96.7 ± 4.3 ^g^	79.0 ± 3.2 ^f^	1.7 ± 0.7 ^a^

Note: Values presented are means of three independent experiments with five replicates in each ± SE (standard error). One-way analysis of variance (ANOVA) was used, and means were compared using a Student’s *t* test with a probability of 95%. Values marked with a similar letter had no significant differences at *p* ≤ 0.05. Embryoids visible to the naked eye were counted in a Petri dish (6 cm in diameter) at 30 days of cultivation. * Mean embryoid numbers per Petri dish, pcs.

**Table 2:** Ploidy level of red cabbage plants regenerated from microspore-derived embryoids.

Accession	No. of Plants	Ploidy Level					
		Diploid		Aneuploid		Tetraploid	
		No.	%	No.	%	No.	%
b.a. 428	4	4	100	0.0	0.0	0	0.0
b.a. 1	8	8	100	0.0	0.0	0	0.0
cv. Gako	30	25	83.3	2	6.7	3	10.0
F1 Red Jewel	16	16	100	0	0.0	0	0.0
b.a. 7-3	30	27	90.0	1	3.3	2	6.7
Total	88	80		3		5	
Mean, %			90.9		3.4		5.7

## Data Availability

Not applicable.
